# Persistent Macular Edema in a Pediatric Vogt-Koyanagi-Harada Disease Patient: A Case Report

**DOI:** 10.7759/cureus.29635

**Published:** 2022-09-26

**Authors:** Eid A Almasoudi, Abubakr S Alzwaihri, Abdullah S Alqahtani

**Affiliations:** 1 College of Medicine, University of Jeddah, Jeddah, SAU; 2 Ophthalmology, King Abdullah International Medical Research Center, Jeddah, SAU; 3 Ophthalmology, King Abdulaziz Medical City, National Guard Hospital, Jeddah, SAU; 4 Ophthalmology/Vitreoretinal and Ocular Oncology Surgery, King Saud Bin Abdulaziz University for Health Sciences, Jeddah, SAU

**Keywords:** uveomeningoencephalitic syndrome, saudi arabia, opthalmology, pediatric, vkhd

## Abstract

Vogt-Koyanagi-Harada disease (VKHD) is a rare multisystemic granulomatous disorder that mainly affects the central nervous system, eyes, inner ears, and skin, basically organs rich with melanocytes. This case report describes an 11-year-old Saudi Arabian female who presented with a six-month history of decrease in vision in both eyes associated with neck pain, right ankle pain, fatigability, and skin depigmentation. Her ophthalmological examination showed visual acuity of 6/30 oculus dextrus (OD) and 6/60 oculus sinister (OS), and her fundoscopic examination revealed vitreous opacity mainly in the right eye. Optical coherence tomography (OCT) demonstrated macular edema along with infiltration and optic edema. She was initially diagnosed as having posterior uveitis and treated with oral prednisone and steroid eye drops. A month later, her ophthalmological examination revealed a rebound of macular edema. Dosages of steroid and adalimumab injection were raised, and azathioprine was added. Her left macular edema was not resolved; therefore, an aflibercept injection was added.

A differential diagnosis of VKHD needs to be considered. Any patient who presents with posterior uveitis should be screened for VKHD. Physicians and ophthalmologists need to be more aware of VKHD, as it can cause serious complications.

## Introduction

Vogt-Koyanagi-Harada disease (VKHD), previously known as uveomeningoencephalitic syndrome, is a rare multisystemic granulomatous disorder of unknown etiology [1]. It mainly affects people with pigmented skin such as Hispanics, Asians, and people who live in Middle East areas and is generally common in females [1]. The disease mainly affects the central nervous system, eyes, inner ears, and skin, basically organs rich with melanocytes [2]. Vogt-Koyanagi-Harada disease is an autoimmune disorder that was first described in 1906 by Alfred Vogt, who reported a case of whitening eyelashes associated with bilateral iridocyclitis. Years later, Harada documented a series of cases that presented with bilateral retinal detachment and cerebrospinal fluid (CSF) pleocytosis. After that, Koyanagi published an article discussing the relationship between posterior eye involvement and auditory manifestations. The condition was, thus, named the Vogt-Koyanagi-Harada disease [1,3,4].

In 1978, the American Uveitis Society (AUS) proposed the criteria for diagnosing VKHD, which include the absence of any history of ocular trauma or surgery in addition to one of the following four items: bilateral chronic iridocyclitis; posterior uveitis involving multifocal exudative retinal detachments, disc hyperemia or edema, or sunset glow fundus; neurological findings of tinnitus, neck stiffness, or cerebrospinal fluid pleocytosis; and cutaneous manifestations (alopecia, poliosis, or vitiligo) [1,5]. The exact pathophysiology of VKHD is not fully understood; however, autoimmunity against melanocyte-associated antigen is thought to play a fundamental role in the pathogenicity [6]. The disease has four stages: prodromal stage, acute uveitic stage, convalescent stage, and recurrent or chronic stage. These stages vary according to the presentation [1].

Here, we reported a unique case of VKHD that had been treated for posterior uveitis for more than six months. The rarity of this disease made the diagnosis challenging. To the best of our knowledge, we could not find in the literature a case of pediatric VKHD with persistent macular edema after initial treatment with different medications particularly for macular edema, such as corticosteroids, azathioprine, and adalimumab, for a significant portion of time without an obvious response.

## Case presentation

**Figure 1:**
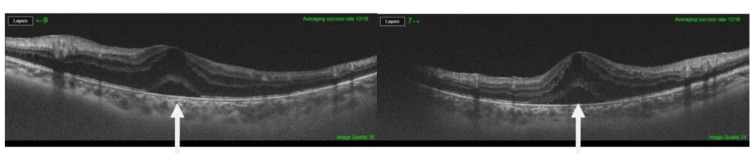
OCT showing macular edema bilaterally and subretinal fluid at the same time (arrows). OCT: optical coherence tomography

**Figure 2:**
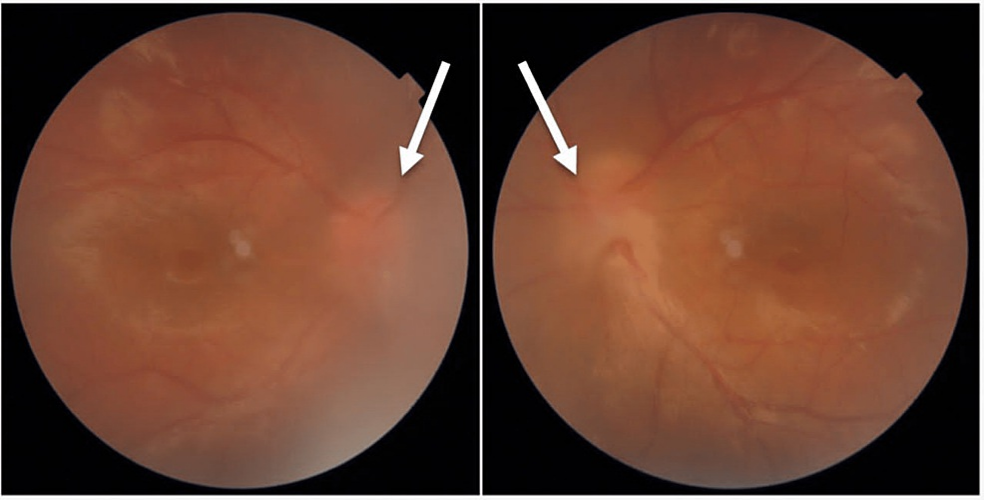
Bilateral papilledema with macular edema in the fundus (arrows).

An 11-year-old Saudi Arabian female presented with a six-month history of decrease in vision in both eyes associated with neck pain, right ankle pain, fatigue, and skin depigmentation. Ophthalmological examination showed visual acuity of 6/30 oculus dextrus (OD) and 6/60 oculus sinister (OS) and a normal anterior segment. Fundoscopic examination revealed vitreous opacity noted mainly in the right eye. In addition, optical coherence tomography (OCT) demonstrated macular edema along with infiltration, optic edema predominantly in the left eye, and bilateral neurosensory detachment (Figures 1, 2). The ankle pain was severe with sudden onset, occurred mainly in the morning, and improved with mobility. Moderate exertion such as climbing stairs seemed to exacerbate her pain. Moreover, there were tinnitus and skin depigmentation areas on her back. Importantly, the condition was not associated with headache, nausea, vomiting, or pain in the eyes. Her past medical history was unremarkable.

**Figure 3:**
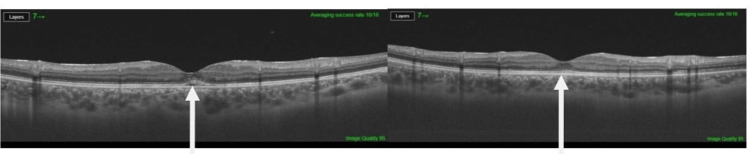
OCT showing improvement of macular edema in both eyes (arrows). OCT: optical coherence tomography

**Figure 4:**
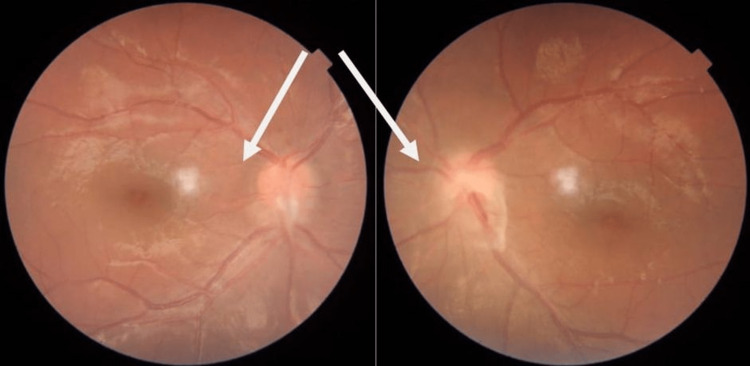
Improvement of the papilledema and macular edema in addition to the remaining fibrotic tissue over both optic nerves (arrows).

There was no previous history of ocular trauma or surgery. She was diagnosed as having posterior uveitis and was started on oral prednisone and steroid eye drops. She was referred to the neurology and rheumatology departments based on her positive family history of multiple sclerosis and rheumatic heart disease. Apart from high intracranial pressure, the laboratory investigations including cerebrospinal fluid (CSF) analysis were otherwise clear; however, she was commenced on 25 mg topiramate tablets twice a day. On follow-up, there was a significant improvement in her condition as evidenced by moderate improvement of the right macular edema with almost complete resolution of the left macular edema, and visual acuity of 6/24 bilaterally. As a result of moderate improvement of the right macular edema, the prednisone dosage was raised. Magnetic resonance imaging (MRI) of the brain, orbits, and spine was done to rule out optic neuritis, transverse myelitis, and space-occupying lesions of the brain that cause compression of optic pathways and was found to be unremarkable. One month after tapering the steroid and shifting to immunosuppressive medications, the ophthalmological examination revealed a rebound of the macular edema. Therefore, the dosages of steroid and adalimumab injection (single dosage every two weeks) were raised, and azathioprine was added because it was considered an insufficient dose or relapse of the disease needs more treatment, which helped improve macular edema. During follow-up after one month, a subcapsular cataract likely due to steroid side effects was noted. In addition, the left macular edema was not resolved; therefore, an aflibercept injection was administered in the left eye, which showed a significant improvement (Figures 3, 4).

The diagnosis of VKHD is challenging; however, the criteria published in the literature can aid in making the appropriate diagnosis (Table 1) and excluding other differential diagnoses such as sarcoidosis and pars planitis. The absence of erythema nodosum, sinusitis, interstitial pneumonitis, pericarditis, myocarditis, interstitial cystitis, and hilar lymph nodes on the chest X-ray and the normal serum calcium exclude sarcoidosis diagnosis. However, a biopsy to look for non-caseating granuloma was not done. Furthermore, since steroids are the mainstay of treatment for sarcoidosis, the diagnosis of sarcoidosis seemed unlikely in a patient who had a rebound and prolonged macular edema after receiving steroids, and therefore, sarcoidosis diagnosis was excluded. Another possible differential diagnosis was pars planitis. Despite the presence of macular edema and snowball in this patient, the characteristic vitreous cell infiltration seen in pars planitis was absent, making this diagnosis unlikely.

**Table 1:** Criteria for diagnosing VKHD. VKHD: Vogt-Koyanagi-Harada disease

Absence of any history of ocular trauma or surgery in addition to one of the following four criteria:
1. Bilateral chronic iridocyclitis
2. Posterior uveitis involving multifocal exudative retinal detachments, disc hyperemia or edema, or sunset glow fundus
3. Neurological findings of tinnitus, neck stiffness, or cerebrospinal fluid pleocytosis
4. Cutaneous manifestations (alopecia, poliosis, or vitiligo)

## Discussion

VKHD must be considered in the differential diagnosis of patients presenting with posterior uveitis [5]. The ophthalmological findings in VKHD most commonly occur bilaterally and usually present days after the prodromal stage. The most common presentation is blurring or loss of vision, nummular chorioretinal depigmented scars, exudative retinal detachments, recurrent or chronic anterior uveitis, “Sugiura sign,” “sunset glow,” neurological manifestations (tinnitus, meningismus, and CSF pleocytosis), and integumentary manifestations (vitiligo, alopecia, and poliosis). The studies highlighted that the pathology may start in one eye before affecting the other. Multifocal exudative retinal detachments in addition to disc hyperemia or edema and sunset glow fundus are characteristics in this stage [1,5,7].

Algahtani et al. described a case of a Saudi Arabian female who was primarily misdiagnosed and treated as having multiple sclerosis. She presented with uveitis and was started on oral steroids with no significant improvement, thus raising the possibility of an alternative diagnosis [8]. In contrast, when oral prednisone was started in our patient, her condition improved initially, and then, a rebound of the macular edema was noted. This may reflect the diagnostic challenges and the importance of applying the diagnostic criteria of VKHD, in any case, presenting with unexplained uveitis.

A complete history and systemic examination along with a full laboratory workup are crucial to exclude other likely diagnoses. These include systemic ophthalmia, lymphoma, multiple sclerosis, tuberculosis, posterior scleritis, sarcoidosis, and uveal effusion syndrome [9]. In this case, the laboratory workup was clear. Therefore, an alternative diagnosis has been later excluded.

Administering high-dose corticosteroids either orally or intravenously as early as possible is a fundamental part of the treatment. The starting dosage is usually 1,000 mg/day intravenously for three days or 1-1.5 mg/kg/day orally, which is then to be tapered within six months. Tapering the dosage within this time is crucial to promote efficacy, reduce the rate of inflammation, prevent complications and recurrence, and decrease disease severity [9,10]. While in our case, the increase in corticosteroids did not prevent the recurrence of macular edema, and subcapsular cataract was noticed as a side effect of corticosteroid usage. Other possible complications of steroids used for long periods were hyperglycemia, cataract, Cushingoid features, osteoporosis, and fractures [11]. Immunosuppressive drugs, such as azathioprine and cyclosporine, can aid in reducing the risk of complications in patients with VKHD [10]. Our patient received a combination of corticosteroids, azathioprine, and adalimumab for a couple of months, which lead to a good outcome [1]. On follow-up, there was a recurrence of macular edema. Based on this, the choice was made for the patient to receive anti-vascular endothelial growth factor (VEGF), 2 mg intravitreal aflibercept injection dosed monthly, after three initial monthly doses in the left eye, which showed a significant improvement. Consequently, the patient’s condition improved significantly.

In general, patients with VKHD have a good prognosis with more than 60% of patients reporting improvement in visual acuity after treatment. The most common complications are choroidal neovascularization, glaucoma, and cataract [1,12].

## Conclusions

VKHD should be considered in any patient presenting with posterior uveitis, neurological symptoms, and skin/multisystem involvement. We consider a case to be representing one of the variations of VKHD with an unusual presentation. Early diagnosis of VKHD can improve visual prognosis. Any patient who presents with posterior uveitis should be screened for VKHD. High-dose corticosteroid is the mainstay of treatment in this condition. Other immunosuppressive medications can be used in case of non-improvement with initial therapy or progressive symptoms, and anti-VEGF should be used if symptoms and signs do not improve.
